# Effects of Curved Wavefronts on Conductor-Backed Reflection-Only Free-Space Material Characterization Techniques

**DOI:** 10.1155/2015/657254

**Published:** 2015-07-15

**Authors:** Raenita A. Fenner, Edward J. Rothwell

**Affiliations:** ^1^Loyola University Maryland, Baltimore, MD 21210, USA; ^2^Michigan State University, East Lansing, MI 48824, USA

## Abstract

A true plane wave is often not physically realizable in a laboratory environment. Therefore, wavefront curvature introduces a form of systematic error into Free-space material characterization methods. Free-space material characterization is important to the determination of the electric permittivity and magnetic permeability of conductor-backed and in situ materials. This paper performs an error analysis of the impact on wavefront curvature on a Free-space method called the *two-thickness* method. This paper compares the extracted electric and magnetic permeability computed with a plane wave versus a line source for a low-loss dielectric and magnetic radar absorbing material. These steps are conducted for TE and TM plane waves and electric and magnetic line sources.

## 1. Introduction

Error analysis is the study and evaluation of uncertainty in measurement [1]. The determination of the amount of propagated error is an essential step for any experimentally determined quantity. Most error analysis is performed to establish the impact of* random error* on an experimentally determined quantity. Random error is inherently present in all measurements and is due to unpredictable fluctuations in reading of measurement apparatus. Error analysis can also be performed to establish the impact of* systematic error*. Systematic error is due to reproducible inaccuracies in the measurement procedure.

Material characterization is the process of determining the relative magnetic permeability, *μ*_*r*_, and electric permittivity, *ϵ*_*r*_, of a material, where *μ* = *μ*_*r*_ *μ*_0_ and *ϵ* = *ϵ*_*r*_ *ϵ*_0_. Accurate knowledge of *ϵ*_*r*_ and *μ*_*r*_ is necessary for many applications which include dielectric resonator antennas [2] and the design of RFID tag antennas [3]. There are many different material characterization methods. One category of material characterization methods is free-space methods. Free-space methods utilize reflection and transmission data from plane wave illumination to determine *μ*_*r*_ and *ϵ*_*r*_ [4]. However, for many applications transmission data cannot be obtained; an example application is for a conductor-backed material sample. When transmission data cannot be obtained, reflection-only free-space methods are required.

A plane wave is defined as a constant-frequency wave whose wavefronts (surfaces of constant phase) are infinite parallel planes of constant amplitude normal to the phase velocity vector [5]. Since in practice a true plane wave cannot be produced, the assumption of plane wave incidence used in free-space methods introduces a source of systematic error to these methods.

The purpose of this paper is to investigate the effect of the plane wave assumption on reflection-only free-space methods. Work has been done in [6] to quantify the impact of the plane wave assumption for free-space methods which use both reflection and transmission, but work has not been done to address the impact of wave curvature on conductor-backed reflection-only free methods. In this paper, the* two-thickness method* is used as a sample test method. To examine the extent of this form of systematic error, the reflection coefficient is calculated due to a nonplanar incident-field wavefront and used in the extraction scheme in the exact manner as the reflection coefficient due to a plane wave. The extracted *ϵ*_*r*_ and *μ*_*r*_ computed using the nonplanar incident-field wavefront reflection coefficients and plane wave reflection coefficients are then compared.

The reflection coefficient due to a 2-dimensional curved wavefront is determined from examination of the canonical problem of a line source above a material slab. Past similar investigations include examination of a two-dimensional problem of a conducting cylinder with a uniform material coating in [7] and examination of whether the late-time component of the field reflected by a planar slab under line-source illumination can be characterized by a natural mode series [8]. In this work, the scattered electric field produced by both electric and magnetic line sources above a conductor-backed material under test (MUT) is computed via the Sommerfeld integral approach and used to determine a reflection coefficient for the case of a nonplanar incident-field wavefront by maintaining Snell's law of reflection. By varying the height of the line source and the MUT thickness, the effect of wavefront curvature on the accuracy of the extracted material properties can be explored for several types of materials.

## 2. Reflection Coefficients due to Electric and Magnetic Line Sources

The analysis necessary for this paper requires derivation of the reflection coefficient due to electric and magnetic line sources. The reflection coefficients for both electric and magnetic line sources are required in order to emulate both perpendicular and parallel polarization of a plane wave. To derive the reflection coefficients, the scattered electric field due the line sources is initially computed.

For simplicity, the derivation steps are listed in detail in Section 2.1 for the electric line source only. The derivation steps for the magnetic line source are similar and are determined by duality; the derivation for the magnetic line source is summarized in Section 2.2.

### 2.1. Electric Line Source

**Figure 1:**
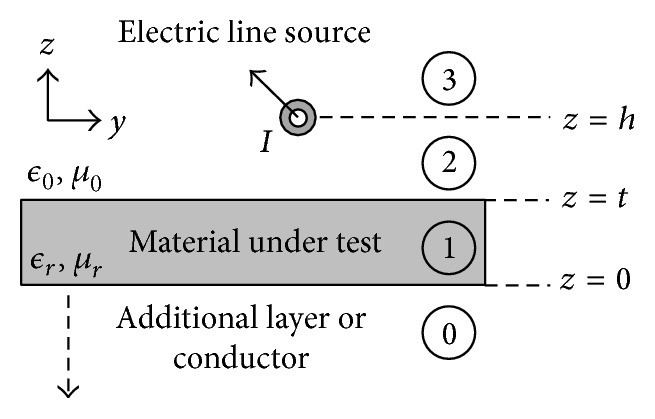
Diagram of the electric line source above a layered slab.

The scattered electric field produced by an electric line source above a conductor-backed MUT is computed via the Sommerfeld integral approach.  Figure 1 depicts the arrangement of the electric line source and MUT used for calculating the scattered electric field.

For the electric line source, the solution approach is as follows:

(1) Write the homogeneous wave equation for the magnetic vector potential, $\nabla^{2}\vec{A}+k^{2}\vec{A}=0$ , in each of the source-free regions depicted in Figure 1. Note that *I* lies on the boundary between two source-free regions of space.
(2) Transform the partial differential equations from step 1 to ordinary differential equations using the Fourier transform and the *z*-invariance of the line source. The magnetic vector potential in the Fourier domain is represented as $\tilde{A}$ .
(3) Assume the proper solutions for $\tilde{A}$ to the ordinary differential equations in step 2 and use boundary conditions for the magnetic and electric fields to solve for the constants of integration.
(4) Find the scattered electric field by using the relationship $\tilde{H}=(1/\mu )\nabla \times \tilde{A}$ or $\tilde{E}=-j\omega \tilde{A}$ .
(5) Use the inverse Fourier transform to find the scattered electric field in the spatial domain.

**Figure 2:**
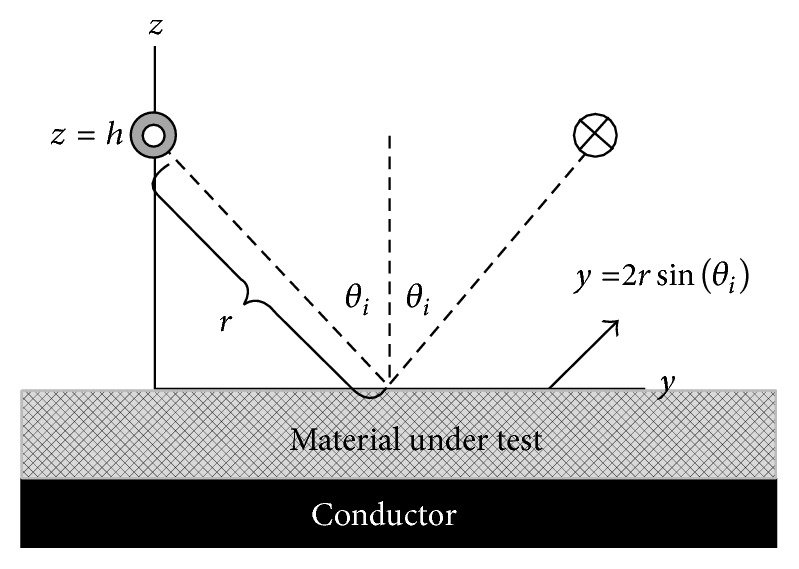
Geometry for reflection coefficient simulations.

The final step in finding the reflection coefficient due to the electric line source is to perform a calibration similar to what would occur in a physical laboratory setup. To calculate the reflection coefficient it is assumed that the electric line source has a height *z* = *h* and an angle *θ*_*i*_ from normal as illustrated in Figure 2. The initial step of the calibration process is to place a PEC plate at the same location as the front layer of the MUT (*z* = *t* in Figure 1) and calculate the scattered electric field supported by the electric line source above the PEC plate, *E*_*x*_^*p*^, using image theory. Next, the reflection coefficient due to the electric line source is calculated via (1), where *E*_*x*_^*p*^ is the scattered field due to the PEC plate alone and *E*_*x*_^*s*^ is the scattered electric field due to the line source and conductor-backed MUT (MUT with a conductor placed at *z* = 0 in Figure 1):

(1) $$\begin{array}{c} \Gamma =-\frac{E_{x}^{s}}{E_{x}^{p}}. \end{array}$$

The final result for the reflection coefficient for the electric line source above the material stack is in (2). Here *H*_0_^(2)^ is the zeroth-order Hankel function of the second kind and *R* is defined in (3). For the solution of both *E*_*x*_^*p*^ and *E*_*x*_^*s*^, the distance and angle of the line source to the MUT are represented by *r* and *θ*_*i*_, respectively, as detailed in Figure 2. Additionally in (2), $k=\omega \sqrt{\mu \epsilon }$ , $k_{0}=\omega \sqrt{\mu_{0}\epsilon_{0}}$ , $p=\sqrt{k_{0}^{2}-k_{y}^{2}}$ , and $q=\sqrt{k-k_{y}^{2}}$ :

(2) Γ=−1+4π1H022k0r·∫0∞e−2jprcos⁡θip+qμ0/μR·cos⁡2kyrsin⁡θidky,

(3) R=1+e−2jqt1−e−2jqt.

### 2.2. Magnetic Line Source

By duality [3], a similar procedure to the one enumerated in Section 2.1 is used to find the scattered electric field due to the magnetic line source.  Figure 1 depicts the arrangement of the magnetic line source and MUT used for calculating the scattered electric field by replacing the infinite electric line source with an infinite magnetic line source. Noted key differences between the procedure in Section 2.1 and the one implemented for the magnetic line source include usage of the electric vector potential, $\vec{F}$ , instead of the magnetic vector potential and usage of $\tilde{E}=-1/\epsilon \nabla \times \tilde{F}$ or $\tilde{H}=-j\omega \tilde{F}$ to compute the scattered electric field. For the calibration procedure used to define the reflection coefficient, (4) is used to compute Γ_*m*_; here *E*_*y*_^*p*^ is the scattered electric field due to a metal plate alone at *z* = 0 in Figure 1 and *E*_*y*_^*s*^ is the scattered electric field due to the magnetic line source and conductor-backed MUT (MUT with a conductor placed at *z* = 0) as in Figure 1 with a magnetic line source instead of an electric line source.

The final result for the reflection coefficient for the magnetic line source above the material stack is in (5). Here *H*_1_^(2)^ is the first-order Hankel function of the second kind and *R* is defined in (3). The terms, *k*, *k*_0_, *p*, *q*, *θ*_*i*_, and *r* are defined in the same way as in Section 2.1. Consider

(4) Γm=−EysEyp,

(5) Γm=−1+4jπ1cos⁡θiH122k0r·∫0∞e−2jprcos⁡θip+qϵ0/ϵRcos⁡2kyrsin⁡θidky.

## 3. Wave Curvature Impact on the Two-Thickness Method

The two-thickness method is a material characterization method which can be implemented via waveguide, probe, or free-space methods. The waveguide implementation of the two-thickness method has been demonstrated by Baker-Jarvis et al. in [9] and an uncertainty analysis performed in [10]. Also, a coaxial-probe implementation of the method is discussed in [11].

**Figure 3:**
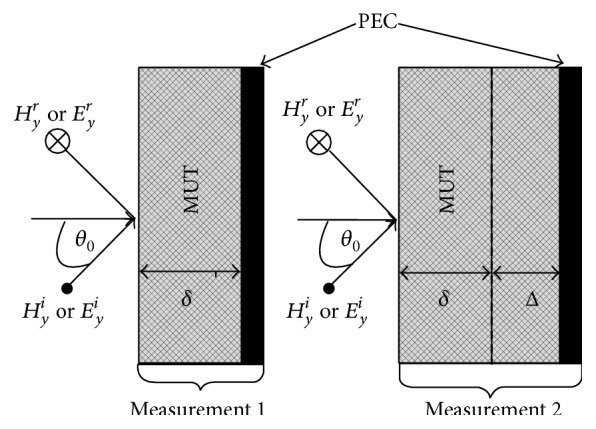
Diagram illustrating the free-space implementation of the two-thickness method.

The free-space implementation of the two-thickness method is utilized by measuring the reflection coefficient for the MUT at two distinct MUT thicknesses [4]. A diagram of the conductor-backed free-space implementation of the two-thickness method is found in Figure 3.

Suppose the two measurements are denoted as measurement 1 and measurement 2. Measurement 1 is made for the MUT at a thickness *δ*. Measurement 2 is made for a MUT thickness *δ* + Δ. The method can be used for any two distinct MUT thicknesses for measurements 1 and 2. However, if Δ = *δ* (thereby making the MUT thickness for measurement 2 exactly* twice* the MUT thickness for measurement 1) the extraction equations are in closed form. A complete treatment on the derivation of the two-thickness method can be found in [12].

Equations (6) and (7) are the extraction equations for the free-space implementation of the two-thickness method for the case when Δ = *δ*. The closed-form equations are only presented here as all of the analyses performed in this paper are on MUT thickness when Δ = *δ*. The extraction equations are dependent on the polarization of the incident wave. Equations (6) are for TM polarized waves and (7) are for TE polarized waves. The terms *Z*_2_ and *k*_*z*2_ are the wave impedance and *z*-component of the wave vector in the MUT and are defined in (8). The terms *Z*_1_^1^ and *Z*_1_^2^, (9), are the free-space wave impedances and are calculated with the measured reflection coefficients from measurements 1 and 2. Lastly, *Z*_0_ = *η*_0_cos⁡*θ*_*i*_ for TM polarization and *Z*_0_ = *η*_0_/cos⁡*θ*_*i*_ for TE polarization, where $\eta_{0}=\sqrt{\mu_{0}{/\epsilon }_{0}}$ :

(6) ϵr=kz2η0k0Z2,μr=kz22+k02sin2⁡θ0k02ϵr,

(7) μr=kz2Z2k0η0,ϵr=kz22+k02sin2⁡θ0k02μr,tan⁡kz2δ=−1−2Z11Z12,Z12=Z01+Γ21−Γ2.

(8) Z22=Z1122Z11/Z12−1,tan⁡kz2δ=−1−2Z11Z12,Z12=Z01+Γ21−Γ2.

(9) Z11=Z01+Γ11−Γ1,Z12=Z01+Γ21−Γ2.

Analysis in this paper is performed on a hypothetical implementation of the two-thickness method. To see the impact of wave curvature on the two-thickness method, *ϵ*_*r*_ and *μ*_*r*_ are extracted with the reflection coefficients due to the electric and magnetic line sources given in Section 2. These reflection coefficients are used in the two-thickness extraction scheme in the exact manner as the reflection coefficients computed with plane waves. The arrangement of the line source for analysis is portrayed in Figure 2. The line source distance, *h* in Figure 2, is varied while the incidence angle and MUT thickness are held constant. Then *ϵ*_*r*_ and *μ*_*r*_ that are extracted as line source distance is increased are compared to their nominal values. This procedure is conducted for Plexiglas (a low-loss dielectric) and a representative magnetic radar absorbing material (MagRAM). A MagRAM is a lossy magnetic material often used in shielding and radar cross section reducing applications. This procedure is conducted for both TE and TM polarization. The angle of incidence is set to *θ* = 40° and the frequency is set to 3 GHz. The values for the angle of incidence, *θ*, and the operating frequency are chosen such that as little random error is introduced into the extracted *ϵ*_*r*_ and *μ*_*r*_ as possible based upon error analysis performed in [6].

## 4. Results

### 4.1. Plexiglas

Analysis is initially performed on a Plexiglas sample. The permittivity value used for Plexiglas is taken from measurements performed in [13] and is thus *ϵ*_*r*_ = 2.65 − *j*0.0076; Plexiglas is a nonmagnetic material; therefore *μ*_*r*_ = 1.0 − *j*0.0.

Initially, it is worthwhile to compare the reflection coefficients calculated with plane waves versus the reflection coefficients calculated with the electric and magnetic line sources. The reflection coefficients calculated with the plane wave assumption are in Table 1.

Table 2 contains the reflection coefficients calculated with the electric and magnetic line sources for different line source distance (LSD) or *r* in Figure 3 in meters. In Table 1 and the remaining tables in this paper, Γ_1_ denotes the reflection coefficient measured when the MUT is *δ* mil thick and Γ_2_ denotes the reflection coefficient measured when the MUT is *δ* + Δ mil thick. In this section, the MUT thicknesses are chosen as 0.1016 cm and 0.2032 cm. Again, the second MUT thickness is chosen to be* twice* the first MUT thickness (i.e., *δ* + Δ) such that extraction equations for the two-thickness method are in closed form.

Comparison of Tables 1 and 2 shows excellent agreement between the reflection coefficients calculated with the line sources and plane wave assumptions. Observation of the reflection coefficients with the TM polarized plane waves and the reflection coefficients calculated with the magnetic line source shows a maximum percent deviation of 1.0% and 0.02%, respectively, for the magnitude and phase of Γ_1_ and Γ_2_. For the TE polarized wave and electric line source, percent deviations of 0.03% and 0.01% for the phase of Γ_1_ and Γ_2_ are observed. No deviation for up to four digits is observed for the magnitude of the reflection coefficients in the case of the TE polarized plane waves and electric line source.

**Figure 4:**
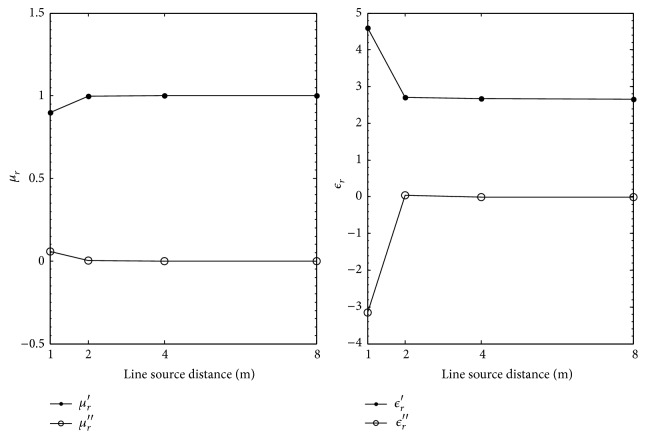
Extracted *μ*_*r*_ and *ϵ*_*r*_ for Plexiglas versus electric line source distance in meters.

Table 3 and Figure 4 show the extracted values of *ϵ*_*r*_ and *μ*_*r*_ using the reflection coefficients calculated with the electric line source using the two-thickness method for increasing line source distance. When the line source is 1 m from the top of the MUT surface, the worst extractions for *ϵ*_*r*_ and *μ*_*r*_ are observed. At 1 m, percent deviations of 73.0% and 99.0% for the real and imaginary parts of *ϵ*_*r*_ are observed. A percent deviation of 10% is computed for the real part of *μ*_*r*_. The percent error for the imaginary part of *μ*_*r*_ is not computed as it is impossible to calculate since *μ*_*r*_′′ = 0.0 for Plexiglas. Observation of Figure 4 shows that better extractions are achieved as line source distance is increased. At 8 m, the percent error between the nominal and extracted values for *ϵ*_*r*_ is 0.3% and 15.0%, respectively, for the real and imaginary parts; for the real part of *μ*_*r*_, a 0.0% percent deviation is computed.

**Figure 5:**
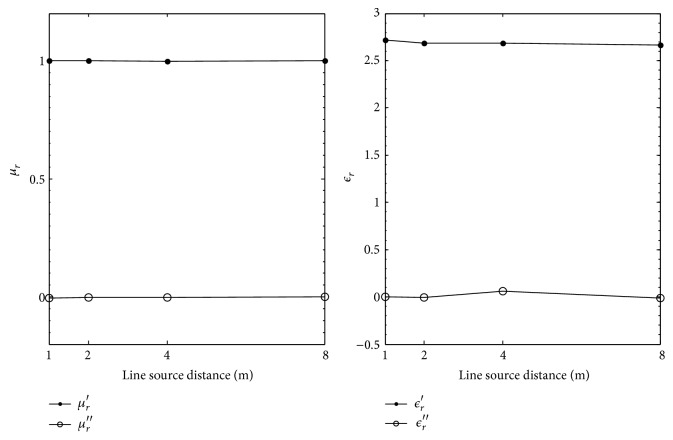
Extracted *μ*_*r*_ and *ϵ*_*r*_ for Plexiglas versus magnetic line source distance in meters.

Table 4 and Figure 5 show the extracted *ϵ*_*r*_ and *μ*_*r*_ with the reflection coefficients calculated with a magnetic line source. Just as the case of the electric line source, the worst extraction is when the line source is 1 m above the MUT. At this distance, the percent errors between the nominal and extracted *ϵ*_*r*_ and *μ*_*r*_ are 2.2% and 89% for the real and imaginary parts of *ϵ*_*r*_ and 0.02% for the real part of *μ*_*r*_. The percent errors are skewed for the imaginary part of *ϵ*_*r*_ because the nominal value of *ϵ*_*r*_ is small. Again, as with the case of the electric line source, Figure 5 shows that the increase of the magnetic line source distance from the MUT yields better agreement between the nominal and extracted *ϵ*_*r*_ and *μ*_*r*_. At 8 m, the percent errors difference between the nominal and extracted real and imaginary parts of *ϵ*_*r*_ is 0.5% and 46%. For the real part of *μ*_*r*_, a percent error of 0.09% is calculated. Although the percent error at 8 m is larger than the percent error at 1 m for *μ*_*r*_′, this is considered to be relatively unimportant as all the extracted *μ*_*r*_ values are close to unity.

Comparison of the extracted *ϵ*_*r*_ and *μ*_*r*_ for the electric and magnetic line sources in Tables 3 and 4 shows much better agreement to the nominal material parameters for the magnetic line source. Note that for both line sources there was great agreement to the plane wave reflection coefficients. The extraction for the magnetic line source is better than the extraction with the electric line explained by the fact that the TM extraction equations are less susceptible to fluctuations in the reflection coefficient than the TE equations for low-loss dielectrics. For a conductor-backed low-loss dielectric like Plexiglas, the reflection coefficient is close to −1. This causes cancellation effects in the calculation in *Z*_2_ in (8). The cancellation effects in *Z*_2_ have more of an impact on the extraction equations for TE polarized waves in (7) because *Z*_2_ is in the numerator for *μ*_*r*_; thus, the cancellation effects propagate into the solution. For TM polarization, *Z*_2_ is in the denominator of *ϵ*_*r*_ which negates the impact of the cancellation effects.

An important remark must be made as to the variation in the extracted *ϵ*_*r*_ and *μ*_*r*_ from their nominal values. In consideration of the excellent agreement of the reflection coefficients calculated with both line sources and polarized plane waves, it would be expected that extraction of *ϵ*_*r*_ and *μ*_*r*_ would yield good results for all of the cases simulated. This phenomenon can be explained by the statistical dependence of extracted *ϵ*_*r*_ and *μ*_*r*_ upon variation of measured reflection coefficients (i.e., standard deviation due to magnitude and phase of the reflection coefficient). Although the standard deviation is generally a statistical measure of random error due to random fluctuations in a dependent variable, the fluctuations here are due to the wavefront curvature of the electric and magnetic line sources.

### 4.2. MagRAM

Again, the material parameters for the MagRAM sample which are determined by measurement in [13] are *ϵ*_*r*_ = 10.65 − *j*1.50 and *μ*_*r*_ = 1.65 − *j*0.9. Just as the case of Plexiglas, it is worthwhile to compare the reflection coefficients calculated with plane waves versus the reflection coefficients calculated with the electric and magnetic line sources. The reflection coefficients calculated with the plane wave assumption are in Table 6.  Table 5 contains the reflection coefficients calculated with the electric and magnetic line sources for different line source distances (LSD) in meters. Again, Γ_1_ denotes the reflection coefficient measured when the MUT is *δ* = 0.1016 cm thick and Γ_2_ denotes the reflection coefficient measured when the MUT is *δ* + Δ = 0.2032 cm thick.

Comparison of Tables 5 and 6 shows excellent agreement between the reflection coefficients calculated with the line sources and plane wave assumptions. Observation of the reflection coefficients with the TM polarized plane waves and the reflection coefficients calculated with the magnetic line source shows a maximum percent error of 0.05% and 0.12% for the phase and magnitude, respectively, of both Γ_1_ and Γ_2_. A maximum percent error of 4.9% and 0.14% is observed for the phase and magnitude of Γ_1_ and Γ_2_ for the electric line source and TE polarized plane wave.

**Figure 6:**
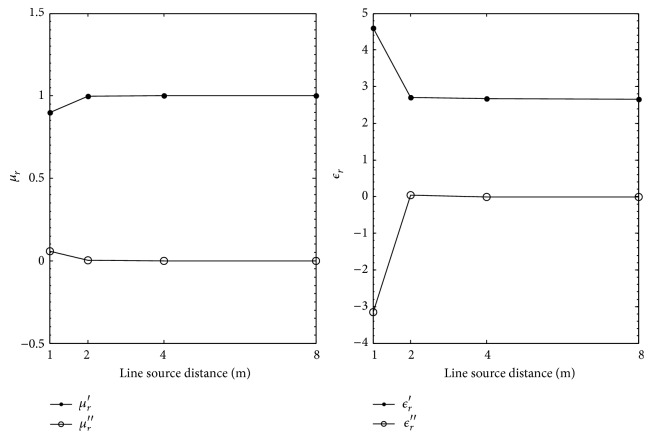
Extracted *μ*_*r*_ and *ϵ*_*r*_ for MagRAM versus electric line source distance in meters.

Table 7 and Figure 6 show the extracted values of *ϵ*_*r*_ and *μ*_*r*_ using the reflection coefficients calculated with the electric line source. There is very little variation in the extracted values. At 1 m, percent deviations of 0.19% and 0.78% are observed for the real and imaginary parts of *μ*_*r*_; 0.54% and 2.3% are observed for the real and imaginary parts of *ϵ*_*r*_. As the line source distance is increased better extractions are achieved as it appears that the extracted values are converging toward the nominal values as shown in Figure 6. At 8 m, the percent deviation between the nominal and extracted values for *ϵ*_*r*_ is 0.07% and 0.26%, respectively, the real and imaginary parts; for the real and imaginary part of *μ*_*r*_, percent deviations of 0.02% and 0.10% are computed. The excellent agreement between the extracted and nominal values for *ϵ*_*r*_ and *μ*_*r*_ for the remaining line sources distances of 2 m and 4 m is also noted.

**Figure 7:**
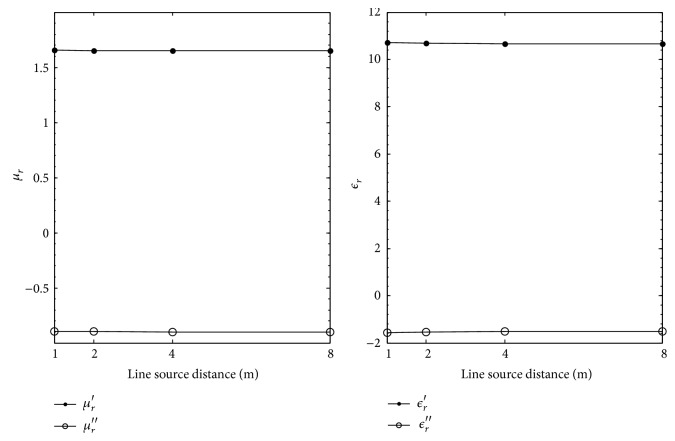
Extracted *μ*_*r*_ and *ϵ*_*r*_ for MagRAM versus magnetic line source distance in meters.

Table 8 and Figure 7 show the extracted *ϵ*_*r*_ and *μ*_*r*_ with the reflection coefficients calculated with the magnetic line source. Similar to the case with the electric line source, Figure 7 shows the extracted *ϵ*_*r*_ and *μ*_*r*_ of the MagRAM with the magnetic line source having very little variation with increase of line source distance. A maximum percent deviation of 4.5% is calculated for all of the extracted values.

Comparison of the extractions performed on the MagRAM sample and the Plexiglas sample in Section 4.1 shows that the extractions made on the MagRAM sample are generally more accurate than the extractions made on the Plexiglas sample. More specifically, the extractions performed with the electric line source on the Plexiglas sample show the worst performance. In Section 4.1, it is explained that the TE extraction equations for the two-thickness method are sensitive to cancellation effects for low-loss dielectrics. The accurate extraction with the MagRAM sample reinforces this explanation because the extraction equations are immune to cancellation effects. In general, lossy materials which are conductor backed will be immune to cancellation effects as the waves will attenuate as they propagate through the material and the reflection coefficient will differ greatly from that of the conductor backing.

## 5. Conclusions

The impact of wave curvature on free-space material characterization methods has been investigated. The plane wave assumption in free-space material characterization methods can be thought of as a type of systematic error in all free-space methods because a true plane wave is not physically realizable in a laboratory environment.

To test the impact of wave curvature, the Fresnel reflection coefficients for TE and TM polarized waves are replaced with reflection coefficients calculated with electric and magnetic line sources in the two-thickness extraction algorithm. Results showed very little impact on the reflection coefficients or extracted *ϵ*_*r*_ and *μ*_*r*_ values for the MagRAM sample. However, the simulations on Plexiglas show that care must be taken when performing characterization on conductor-backed low-loss dielectrics as these materials do not provide a means for attenuation and the reflection coefficient is dominated by the presence of the conductor backing. This in turn causes the extraction equations to be sensitive to cancellation effects. It is also noted that these cancellation effects will be present for the characterization of *ϵ*_*r*_ alone (in the cases when the material is known to be a dielectric a priori) because the measured reflection coefficient will still be heavily impacted by the conductor backing. It is important to note that the sensitivity described in the Plexiglas characterization is not due to wavefront curvature but is inherent in the extraction of low-loss dielectrics which are conductor backed. Therefore, from the samples simulated it is concluded that wavefront curvature does not introduce a large amount of error into the two-thickness method and similar free-space material characterization methods.

## Figures and Tables

**Table 1:** Reflection coefficients calculated with plane wave assumption (Plexiglas).

Polarization	Γ_1_	Γ_2_
TM	1.002*∠*171.95°	1.0016*∠*163.79°
TE	0.9996*∠*174.43°	0.9992*∠*168.69°

**Table 2:** Reflection coefficients calculated with electric and magnetic line sources (Plexiglas).

LSD (m)	Plexiglas			
	Electric		Magnetic	
	Γ_1_	Γ_2_	Γ_1_	Γ_2_
1	0.9996*∠*174.37°	0.9999*∠*168.68°	1.0023*∠*171.97°	1.0016*∠*163.76°
2	0.9997*∠*174.37°	0.9999*∠*168.67°	1.0001*∠*171.92°	1.0002*∠*163.76°
4	0.9998*∠*174.37°	0.9997*∠*168.68°	1.0000*∠*171.92°	1.0002*∠*163.76°
8	0.9999*∠*174.37°	0.9998*∠*168.68°	1.0005*∠*171.92°	0.9999*∠*163.77°

**Table 3:** Extracted *μ*_*r*_ and *ϵ*_*r*_ calculated with electric line source reflection coefficients (Plexiglas).

LSD (m)	*μ*_*r*_′	*μ*_*r*_′′	*ϵ*_*r*_′	*ϵ*_*r*_′′
1	0.8980	0.0588	4.6028	−3.1556
2	0.9971	0.0015	2.6979	0.0409
4	1.0000	−0.0010	2.6667	−0.0059
8	1.0000	−0.0005	2.6582	−0.0064

**Table 4:** Extracted *μ*_*r*_ and *ϵ*_*r*_ with magnetic line source reflection coefficients (Plexiglas).

LSD (m)	*μ*_*r*_′	*μ*_*r*_′′	*ϵ*_*r*_′	*ϵ*_*r*_′′
1	1.0002	−0.0040	2.7171	−0.0008
2	1.0001	−0.0020	2.6836	−0.0043
4	0.9977	−0.0036	2.6861	0.0625
8	0.9991	0.0006	2.6639	−0.0111

**Table 5:** Reflection coefficients calculated with electric and magnetic line sources (MagRAM).

LSD (m)	MagRAM			
	Electric		Magnetic	
	Γ_1_	Γ_2_	Γ_1_	Γ_2_
1	0.9112*∠*170.65°	0.8070*∠*160.66°	0.8559*∠*164.37°	0.7068*∠*147.59°
2	0.9115*∠*170.64°	0.8077*∠*160.62°	0.8556*∠*164.39°	0.7066*∠*147.63°
4	0.9117*∠*170.63°	0.8080*∠*161.23°	0.8554*∠*164.40°	0.7064*∠*147.65°
8	0.9118*∠*170.63°	0.8082*∠*168.63°	0.8553*∠*164.44°	0.7063*∠*147.66°

**Table 6:** Reflection coefficients calculated with plane wave assumption (MagRAM).

Polarization	Γ_1_	Γ_2_
TE	0.9112*∠*170.65°	0.8070*∠*160.66°
TM	0.8560*∠*164.37°	0.7072*∠*147.59°

**Table 7:** Extracted *μ*_*r*_ and *ϵ*_*r*_ calculated with electric line source (MagRAM).

LSD (m)	*μ*_*r*_′	*μ*_*r*_′′	*ϵ*_*r*_′	*ϵ*_*r*_′′
1	1.6468	−0.9071	10.7081	−1.4652
2	1.6484	−0.9036	10.6792	−1.4828
4	1.6492	−0.9018	10.6644	−1.4916
8	1.6496	−0.9009	10.6576	−1.4961

**Table 8:** Extracted *μ*_*r*_ and *ϵ*_*r*_ calculated with magnetic line source (MagRAM).

LSD (m)	*μ*_*r*_′	*μ*_*r*_′′	*ϵ*_*r*_′	*ϵ*_*r*_′′
1	1.6538	−0.8944	10.7107	−1.5677
2	1.6518	−0.8972	10.6820	−1.5354
4	1.6510	−0.8986	10.6655	−1.5201
8	1.6504	−0.8994	10.6569	−1.5066
